# Clinical Outcomes of Diode Laser Ablation Versus Diode Laser Excision in the Management of Oral Leukoplakia: An Observational Study

**DOI:** 10.7759/cureus.113690

**Published:** 2026-07-30

**Authors:** Nisha Dua, Nisha Singh, Rajesh K Gupta, Kamakshi Uppal, Archana Sharma, Manpreet Cheema

**Affiliations:** 1 Department of Oral Medicine and Radiology, Swami Devi Dyal Hospital and Dental College, Panchkula, IND

**Keywords:** disease recurrence, dysplasia, laser therapy, leukoplakia, wound healing

## Abstract

Background: Oral leukoplakia is the most common potentially malignant disorder of the oral mucosa (OM). Although diode laser therapy offers a minimally invasive alternative to conventional scalpel excision, the comparative clinical outcomes of diode laser ablation and diode laser excision remain inadequately characterized, particularly regarding postoperative recovery and recurrence risk. Materials and methods: This prospective comparative observational study included 60 patients with histopathologically confirmed homogeneous oral leukoplakia who underwent diode laser ablation (n = 30) or diode laser excision (n = 30) based on the surgeon's clinical decision. Postoperative outcomes, including wound healing, pain, bleeding, and swelling, were assessed at one, three, and five weeks. Recurrence and functional disturbances were assessed at the six-month follow-up. A three-way mixed repeated-measures ANOVA was used to analyze longitudinal changes in wound healing, pain, bleeding, and swelling according to treatment modality, dysplasia grade, and follow-up time, whereas chi-square tests were used to compare recurrence and functional outcomes. Results: Laser ablation demonstrated significantly superior early postoperative outcomes compared to excision at weeks 1 and 3 across all parameters, including healing, pain, bleeding, and swelling (all p < 0.001), regardless of dysplasia grade. By week 5, wound healing, pain, and swelling were comparable between the treatment groups except bleeding. Dysplasia grade significantly influenced healing (p = 0.004) but not other parameters. Importantly, the overall recurrence rate was significantly higher after ablation than that after excision (26.7% vs. 6.7%, p = 0.038), with the most pronounced difference observed in the mild dysplasia subgroup (46.7% vs. 0.0%, p = 0.006). Conclusions: Diode laser ablation offers superior early postoperative recovery with reduced pain, bleeding, swelling, and accelerated healing compared to excision. However, this short-term benefit is offset by significantly higher recurrence rates, particularly in lesions with mild dysplasia. These findings suggest that a dysplasia-stratified treatment approach may be considered for the management of homogeneous oral leukoplakia. Based on the findings of the present observational study, laser ablation may offer advantages for non-dysplastic lesions, whereas laser excision may reduce recurrence in lesions with mild epithelial dysplasia.

## Introduction

Oral leukoplakia, recognized as the most prevalent potentially malignant disorder of the oral mucosa (OM), necessitates timely clinical intervention to mitigate the significant risk of malignant transformation [1,2]. Oral leukoplakia, recognized as the most prevalent potentially malignant disorder of the OM, carries a reported malignant transformation rate ranging from approximately 1% to 9% [1]. While conventional surgical management, primarily through scalpel excision, remains the historical gold standard because of its ability to provide intact tissue specimens for comprehensive histopathological examination, it is frequently associated with notable postoperative morbidity, including significant patient discomfort, inflammation, and protracted healing times, which can affect the overall patient quality of life [2,3]. Consequently, advanced laser systems have emerged as a significant paradigm shift in the management of oral precancerous lesions, offering a minimally invasive approach characterized by enhanced intraoperative hemostasis, reduced surgical field contamination, and improved wound-healing outcomes [3,4].

Among these diverse technologies, the diode laser, in particular, is increasingly adopted in routine clinical practice because of its inherent portability and accessibility and has demonstrated efficacy in the management of homogeneous leukoplakia [5,6]. However, the clinical decision-making process between diode laser excision, which facilitates definitive histopathological margin evaluation, and diode laser ablation, which is often preferred because of its procedural speed and reduced tissue trauma, remains a topic of critical discussion, particularly regarding long-term recurrence rates and nuanced patient-reported outcomes [2,7]. While ablation offers a streamlined, patient-friendly approach, particularly suitable for wider lesions or anatomical sites where traditional surgical excision may be technically challenging, concerns persist regarding the inability to perform a detailed histopathological margin assessment of vaporized tissue, highlighting a significant trade-off between procedural convenience and diagnostic reliability [7,8].

Furthermore, the unpredictable recurrence of oral leukoplakia remains a substantial clinical hurdle, with rates varying widely across diverse studies, emphasizing the urgent need for a deeper understanding of how specific laser surgical modalities influence the long-term risk of recurrence and the potential for disease progression [1,9]. Despite the well-documented advantages of diode laser therapy in reducing postoperative morbidity, prospective head-to-head comparisons of diode laser ablation and diode laser excision performed using identical laser wavelength, power settings, and treatment protocols remain limited. Consequently, the relative effects of these two techniques on postoperative recovery and recurrence have not been fully established [3,5].

This prospective comparative study aimed to compare postoperative recovery, including wound healing, pain, bleeding, and swelling, and the six-month recurrence rate following diode laser ablation and diode laser excision performed using standardized diode laser parameters in patients with homogeneous oral leukoplakia. We hypothesized that laser ablation would provide superior early postoperative recovery, whereas laser excision would be associated with a lower recurrence rate.

## Materials and methods

Study design and setting

This was a prospective, comparative, clinical observational study conducted in the Department of Oral Medicine and Radiology, Swami Devi Dyal Dental College, Panchkula, Haryana, from May 2024 to December 2025. Patients with a confirmed diagnosis of homogeneous leukoplakia who opted for diode laser therapy as part of routine clinical care were observed, and their treatment outcomes were compared according to the modality received (ablation vs. excision) and histopathological grade (no dysplasia vs. mild dysplasia). No randomization was performed, and group allocation followed existing clinical decision-making based on lesion characteristics and patient preference.

The study was approved by the Institutional Ethical Committee of Swami Devi Dyal Hospital and Dental College (approval number: SDDHDC/IEC/May2024/013), and written informed consent was obtained from all participants prior to group assignment, in accordance with the Declaration of Helsinki.

Inclusion and exclusion criteria

Patient eligibility was established based on predefined inclusion and exclusion criteria, which are presented in Table 1.

**Table 1:** Inclusion and exclusion criteria for participants

Inclusion criteria	Exclusion criteria
Patients aged ≥18 years	Nonhomogeneous or speckled leukoplakia
Clinical diagnosis of flat homogeneous oral leukoplakia	Moderate-to-severe epithelial dysplasia
Histopathologically confirmed diagnosis of oral leukoplakia	Histopathologically confirmed oral malignancy
No or mild epithelial dysplasia	Previous surgical, laser, or pharmacological treatment for the index lesion
Consented to undergo diode laser treatment	Pregnancy or lactation
Willing to comply with the scheduled follow-up protocol	Uncontrolled systemic diseases like bleeding disorders

Sample size estimation

The sample size was calculated with G*Power software (version 3.1.9.2; Heinrich Heine University, Düsseldorf, Germany) based on the obtained difference in mean swelling scores (effect size 0.65) between the diode laser excision and surgical excision groups reported in a previous comparative study [10], with a mean difference of 1.01 points (pooled deviation = 1.5), 80% power, and a two-sided alpha of 0.05, yielding a minimum of 27 patients per group (total 54 patients), which was rounded up to 30 patients per group (total 60 patients) to account for possible 10% attrition during follow-up.

Patient assignment

This was a prospective observational study; therefore, treatment assignment was not randomized but reflected routine clinical practice. The treating oral and maxillofacial surgeon selected diode laser ablation or diode laser excision after evaluating predefined clinical factors, including lesion size, anatomical location, accessibility, lesion morphology (flat or slightly elevated), and the anticipated feasibility of achieving complete lesion removal with adequate surgical margins while preserving adjacent normal tissue. Patient preference was also incorporated into the final treatment decision after counseling regarding the expected advantages and limitations of both procedures. In general, laser ablation was considered for broad superficial lesions or lesions at anatomically challenging sites where tissue preservation and reduced postoperative morbidity were prioritized. In contrast, laser excision was selected when complete lesion removal with histopathological margin assessment was considered clinically important.

Pre-procedural protocol

All patients underwent tobacco cessation counseling based on the 5A principle (ask, advise, assess, assist, and arrange) [11]. A topical antifungal agent, Candid Mouth Paint (1% w/v; Glenmark Pharmaceuticals Ltd., Mumbai, India), was prescribed for one week prior to biopsy to exclude Candida superinfection as a confounder. The clinical diagnosis of homogeneous oral leukoplakia was established by an experienced faculty of oral medicine according to the World Health Organization (WHO) diagnostic criteria. Homogeneous leukoplakia was defined as a predominantly uniform, thin, flat white plaque with a smooth, fissured, or slightly wrinkled surface that could not be clinically characterized as any other definable oral white lesion [1]. Toluidine blue (Sisco Research Laboratories Pvt. Ltd., Mumbai, India) vital staining was performed to guide biopsy site selection, followed by incisional biopsy for histopathological confirmation and dysplasia grading. To ensure uniform postoperative care and minimize potential confounding, all participants, irrespective of the treatment group, received the same postoperative medication regimen. Chemoprophylaxis with Antoxid-HC Capsules (Dr. Reddy's Laboratories Ltd., Hyderabad, India) was recommended daily for one month. As part of standard practice, topical antifungal therapy with Candid Mouth Paint (1% w/v; Glenmark Pharmaceuticals Ltd., Mumbai, India) was prescribed for ten days prior to laser intervention.

Laser treatment procedures

All procedures were performed under regional infiltration anesthesia administered around the periphery of the lesion. An 810-nm gallium-aluminum-arsenide diode laser system (IndiLase; MEDSOL, Hosur, India), incorporating a DILAS laser diode (DILAS, Mainz, Germany), was used with an output power ranging from 1.5 to 3.0 W, operated in continuous-wave mode. Laser energy was delivered through a flexible 400-µm polyamide optical fiber (MED-Fibers, Inc., Chandler, Arizona, USA) coupled to a handheld applicator. The energy delivered was adjusted according to the dimensions and clinical characteristics of the lesion, with cumulative fluences generally ranging between 1600 and 3200 J/cm². Standard aseptic precautions were followed throughout the procedure, and protective eyewear was used by both the operator and patient.

**Figure 1:**
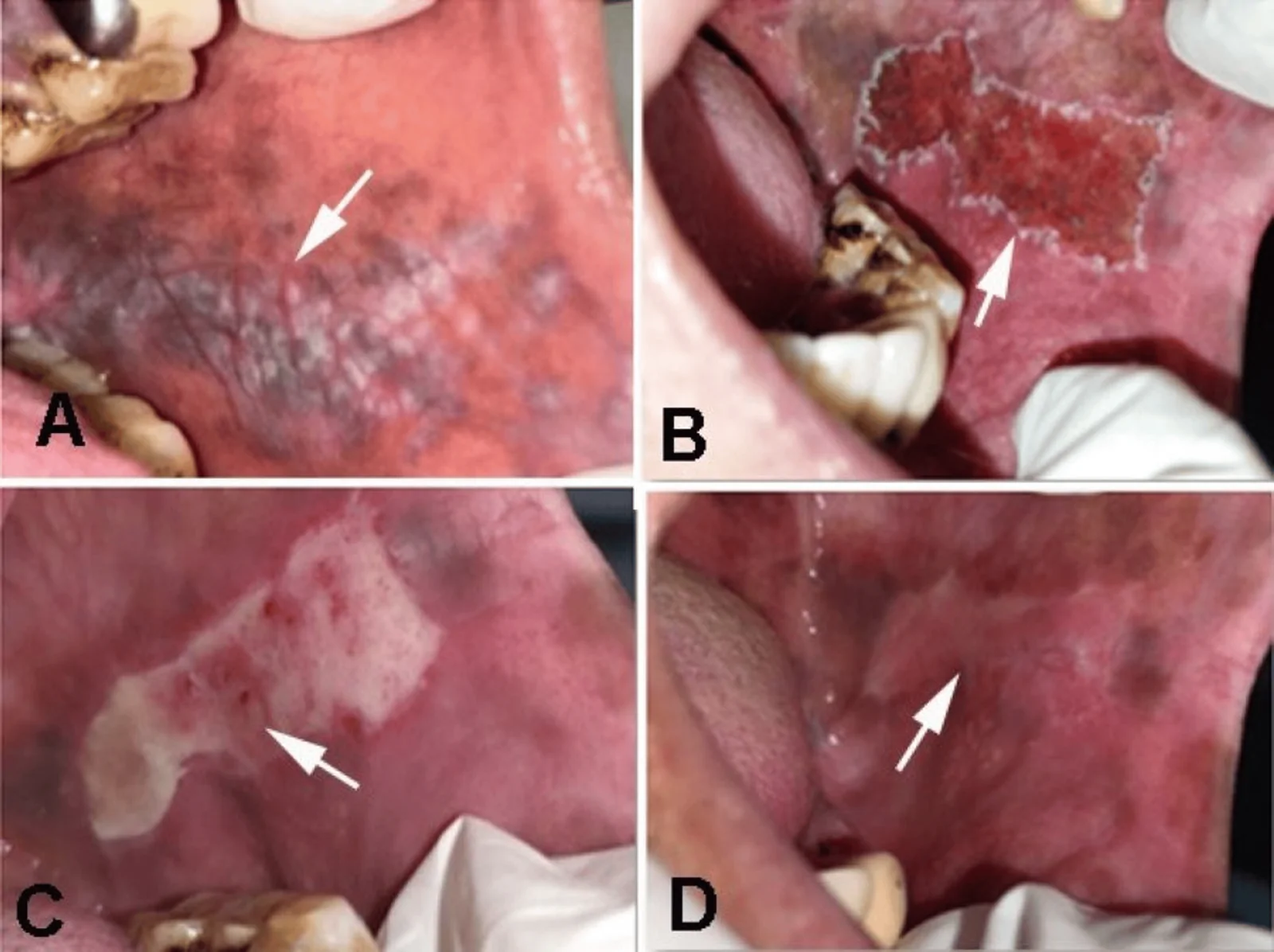
Clinical photographs illustrating laser ablation of homogeneous leukoplakia on buccal mucosa. (A) Preoperative view showing the homogeneous leukoplakic lesion (arrow). (B) One-week postoperative follow-up demonstrating the healing surgical site (arrow). (C) Three-week postoperative follow-up showing progressive epithelialization (arrow). (D) Five-week postoperative follow-up revealing complete healing (arrow) Original image from study data.

In patients managed with laser ablation, the lesion was superficially vaporized along with a narrow margin of clinically normal-appearing mucosa. A peripheral margin of approximately 2-3 mm was included with tissue ablation extending to a depth of approximately 1 mm. Ablation was performed using slow, overlapping brushing strokes from the periphery toward the center of the lesion until complete vaporization of the clinically visible lesion was achieved. Lesions ≤6 mm were treated as single confluent lesions whenever considered clinically appropriate. The intended treatment margin was delineated using a laser prior to tissue vaporization (Figure 1A-1D).

**Figure 2:**
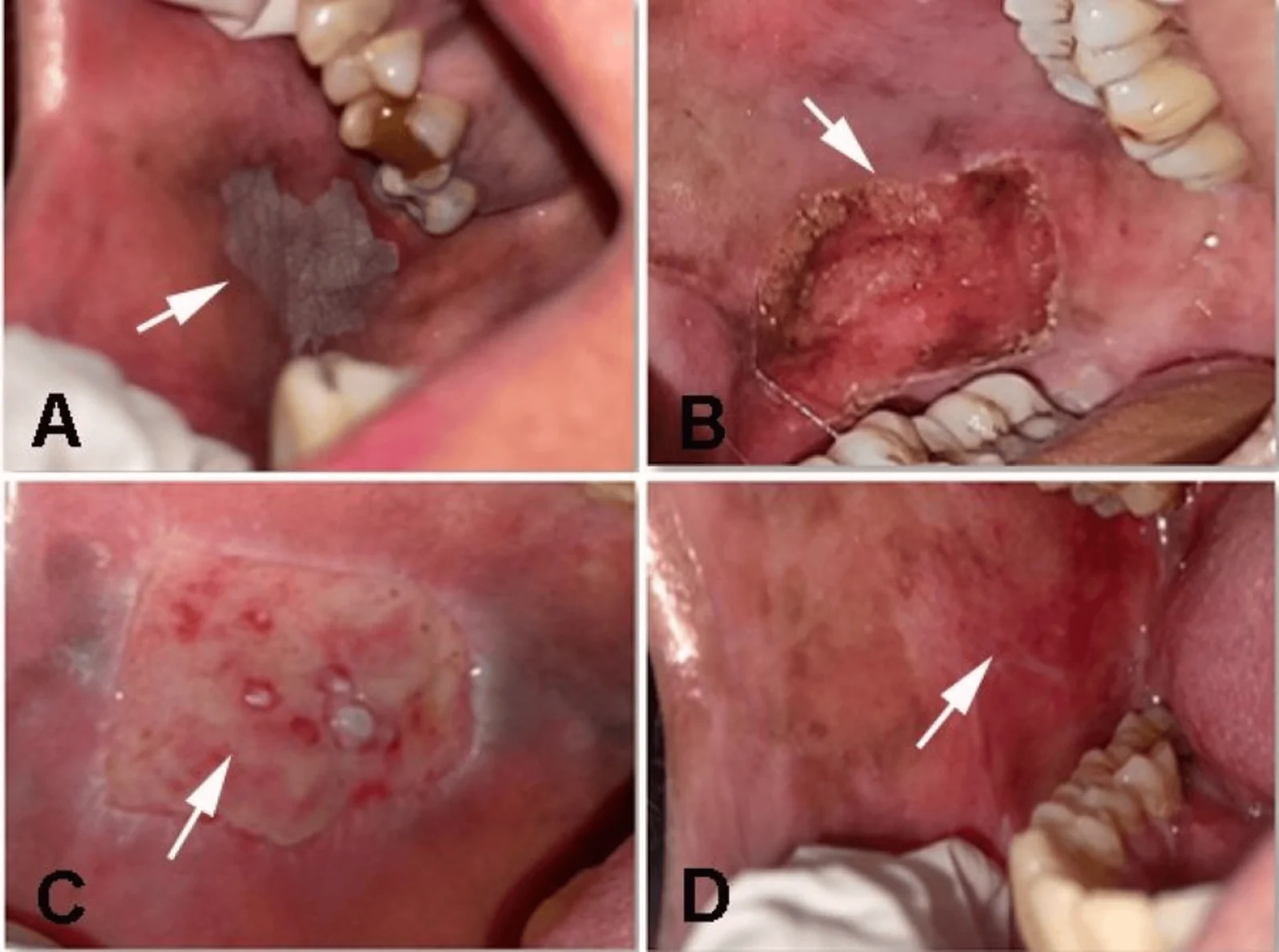
Clinical photographs illustrating laser excision of homogeneous leukoplakia on buccal mucosa. (A) Preoperative view showing the homogeneous leukoplakic lesion (arrow). (B) One-week postoperative follow-up demonstrating the healing surgical site (arrow). (C) Three-week postoperative follow-up showing progressive epithelialization (arrow). (D) Five-week postoperative follow-up revealing complete healing (arrow) Original image from study data.

For laser excision, the clinically visible lesion was completely removed with an approximately 2-3 mm margin of clinically normal-appearing mucosa. The depth of excision was standardized to include the full epithelial thickness and superficial submucosal connective tissue until clinically normal tissue was encountered, while avoiding unnecessary injury to the underlying muscle and adjacent vital structures. The same surgical protocol was applied consistently to all patients by a single experienced oral and maxillofacial surgeon to minimize operator-dependent variability. Circumferential excision was then completed using an undermining technique, maintaining a surgical margin of approximately 3-4 mm where feasible. The excised specimens were routinely submitted for histopathological examination. The surgical wounds were left unsutured for secondary healing. All patients received routine postoperative instructions regarding oral hygiene, wound care, dietary modifications, and prescribed medications, including antibiotics and nonsteroidal anti-inflammatory drugs, according to institutional practice (Figure 2A-2D).

Study outcome measures

The study variables extracted from the clinical records included patient age, sex, anatomical site of the lesion, habit, and type of diode laser treatment performed (ablation or excision). Postoperative outcomes were assessed using standardized clinical indices. Wound healing was evaluated using the Landry, Turnbull, and Howley Healing Index [12], while postoperative pain and bleeding were assessed using a 10-point visual analog scale (VAS) [13]. Postoperative swelling was assessed using the adapted verbal rating scale (VRS) for facial swelling described by Al-Samman and Othman [14], which was based on the VRS methodology originally described by Bosdet et al. [15]. All assessment instruments used are non-proprietary and do not require permission for research use with appropriate citation. Functional disturbances, including impairment of speech, mastication, and tongue mobility, were evaluated using structured clinical assessments during follow-up visits. Long-term outcomes included lesion recurrence, determined by clinical re-examination for the reappearance of leukoplakia at the treated site. All outcome data were obtained from routine follow-up records without influencing patient management.

Follow-up schedule

Patients were evaluated at postoperative weeks 1, 3, and 5 for healing, pain, bleeding, and swelling and at a six-month follow-up visit for recurrence and functional outcomes. No patients were lost to follow-up, and complete outcome data were available for all participants throughout the study.

Statistical analysis

Data were entered into Excel (Microsoft Corp., Redmond, WA, USA) and analyzed using SPSS Statistics version 26.0 (IBM Corp. Released 2019. IBM SPSS Statistics for Windows, Version 26.0. Armonk, NY). Continuous variables (VAS, healing index, VRS scores) were tested for normality using the Shapiro-Wilk test and expressed as mean ± SD or median (IQR), as appropriate. Between-group comparisons (ablation vs. excision) for continuous variables were performed using the independent t-test or Mann-Whitney U test. A three-way mixed (repeated-measures) ANOVA was used to evaluate the main effects of dysplasia grade (between-subject factor), treatment modality (between-subject factor), and follow-up time (within-subject factor), along with their interaction effects on primary outcomes (wound healing, pain, bleeding, and swelling). Categorical variables and secondary outcomes (recurrence, functional disturbance) were compared using the chi-square test or Fisher's exact test, where the expected cell counts were <5. Statistical significance was defined as a two-sided p-value of <0.05.

## Results

The baseline demographic and clinical characteristics of the study participants showed that the ablation and excision groups were well matched, with no statistically significant differences across any parameter (Table 2). The mean age was similar between the groups (p = 0.144), and the duration of tobacco use showed no significant difference (p = 0.093). The lesion size was comparable (p = 0.356), and both groups had a male predominance with a similar sex distribution (p = 0.791). Tobacco habit patterns and lesion site distribution were also evenly matched, with smokeless tobacco being the most common habit and the buccal mucosa being the predominant site in both groups. Histopathological grading showed a comparable distribution between patients with and without dysplasia (p = 0.417), confirming baseline comparability across all measured variables.

**Table 2:** Baseline demographic and clinical characteristics of study participants (N = 60) Data are presented as mean ± SD for continuous variables and frequency (percentage) for categorical variables. Continuous variables were compared using an independent-samples Student's t-test, whereas categorical variables were compared using Pearson's chi-square (χ²) test. Statistical significance was set at p < 0.05. SD: standard deviation

Variable		Ablation (n = 30)	Excision (n = 30)	Test value	p-value
Age (years)	Mean ± SD	48.10 ± 9.20	44.50 ± 9.60	t = 1.48	0.144
Duration of tobacco use (years)	Mean ± SD	10.80 ± 4.90	13.00 ± 5.10	t = -1.71	0.093
Lesion size (cm)	Mean ± SD	1.92 ± 0.64	2.06 ± 0.55	t = -0.93	0.356
Sex, n (%)	Male	18 (60.0)	19 (63.3)	χ² = 0.07	0.791
	Female	12 (40.0)	11 (36.7)		
Tobacco habit type, n (%)	Both	7 (23.3)	5 (16.7)	χ² = 0.70	0.706
	Smokeless	13 (43.3)	16 (53.3)		
	Smoking	10 (33.3)	9 (30.0)		
Lesion site, n (%)	Buccal mucosa	14 (46.7)	14 (46.7)	χ² = 0.62	0.892
	Floor of mouth	3 (10.0)	3 (10.0)		
	Labial mucosa	5 (16.7)	7 (23.3)		
	Tongue	8 (26.7)	6 (20.0)		
Histopathological grade, n (%)	No dysplasia	21 (70.0)	18 (60.0)	χ² = 0.65	0.417
	Mild dysplasia	9 (30.0)	12 (40.0)		

This descriptive table presents the longitudinal evolution of primary outcome measures across the three follow-up time points stratified by treatment group and dysplasia grade. A consistent pattern emerged across healing, pain, and bleeding scores, with progressive improvement from week 1 to week 5 in both treatment groups and dysplasia grades (Table 3). The ablation group consistently demonstrated numerically lower (better) scores at all time points than the excision group, irrespective of dysplasia status.

**Table 3:** Descriptive analysis of outcome variables at multiple time intervals Data are presented as mean ± SD for healing, pain, and bleeding scores and as median (IQR) for swelling. SD: standard deviation, IQR: interquartile range

Parameters	Dysplasia	Follow-up	Ablation (n = 30)	Excision (n = 30)
Healing score (mean ± SD)	No dysplasia	Week 1	2.71 ± 0.54	3.48 ± 0.41
		Week 3	1.63 ± 0.41	2.10 ± 0.50
		Week 5	1.15 ± 0.19	1.35 ± 0.38
	Mild dysplasia	Week 1	2.93 ± 0.35	3.72 ± 0.57
		Week 3	2.09 ± 0.64	2.33 ± 0.49
		Week 5	1.23 ± 0.21	1.25 ± 0.24
Pain score (mean ± SD)	No dysplasia	Week 1	4.76 ± 1.06	6.35 ± 1.07
		Week 3	2.81 ± 0.96	4.27 ± 1.07
		Week 5	1.54 ± 0.85	2.10 ± 0.91
	Mild dysplasia	Week 1	4.78 ± 0.61	6.23 ± 1.03
		Week 3	3.17 ± 0.89	3.95 ± 0.93
		Week 5	2.02 ± 1.07	2.34 ± 0.62
Bleeding score (mean ± SD)	No dysplasia	Week 1	2.80 ± 1.22	5.44 ± 1.12
		Week 3	2.01 ± 1.15	2.88 ± 0.49
		Week 5	1.24 ± 0.66	1.62 ± 0.94
	Mild dysplasia	Week 1	2.75 ± 0.96	5.19 ± 1.21
		Week 3	1.79 ± 0.51	3.43 ± 0.80
		Week 5	1.35 ± 0.64	1.75 ± 0.64
Swelling score (median (IQR))	No dysplasia	Week 1	2.0 (1.0-2.0)	3.0 (2.0-3.0)
		Week 3	1.0 (1.0-1.5)	2.0 (2.0-2.0)
		Week 5	1.0 (0.0-1.0)	1.0 (1.0-1.0)
	Mild dysplasia	Week 1	2.0 (2.0-2.5)	3.0 (3.0-3.0)
		Week 3	1.0 (1.0-2.0)	2.0 (1.5-2.0)
		Week 5	1.0 (1.0-1.0)	1.0 (1.0-1.0)

The three-way mixed ANOVA revealed that the treatment modality (ablation vs. excision) had a significant main effect across all outcome parameters (all p < 0.001), confirming the superiority of ablation for early postoperative recovery (Table 4). Time demonstrated the most substantial effect (η²p = 0.77-0.87), indicating that all outcomes improved significantly over the follow-up period. Significant treatment × time interactions were observed for all parameters (all p < 0.001), suggesting that the rate of improvement differed between the treatment groups, with ablation showing steeper early recovery. Dysplasia grade had a significant independent effect only on healing (p = 0.004), with non-dysplastic lesions healing faster but no effect on pain, bleeding, or swelling. None of the three-way interactions reached significance, indicating consistent treatment effects across dysplasia grade.

**Table 4:** Three-way mixed ANOVA for outcome variables Three-way mixed ANOVA was performed. Time was treated as the repeated-measures factor, whereas treatment and dysplasia grade were between-subject factors. Effect size is presented as partial eta squared (η²p). *p < 0.05 values indicate statistical significance. df: degrees of freedom, ANOVA: analysis of variance

Parameter	Source of variation	F-value	df	p-value	Effect size
Healing	Dysplasia grade (no vs. mild)	9.13	1, 56	0.004*	0.14
	Treatment (ablation vs. excision)	44.63	1, 56	<0.001*	0.44
	Dysplasia × treatment	1.05	1, 56	0.311	0.02
	Time	408.46	2, 112	<0.001*	0.87
	Dysplasia × time	3.17	2, 112	0.026*	0.05
	Treatment × time	12.45	2, 112	<0.001*	0.18
	Dysplasia × treatment × time	0.41	2, 112	0.744	0.07
Pain	Dysplasia grade (no vs. mild)	0.36	1, 56	0.552	0.01
	Treatment (ablation vs. excision)	41.95	1, 56	<0.001*	0.43
	Dysplasia × treatment	1.16	1, 56	0.286	0.02
	Time	347.15	2, 112	<0.001*	0.86
	Dysplasia × time	0.80	2, 112	0.496	0.01
	Treatment × time	8.56	2, 112	<0.001*	0.13
	Dysplasia × treatment × time	0.45	2, 112	0.720	0.01
Bleeding	Dysplasia grade (no vs. mild)	0.16	1, 56	0.691	0.01
	Treatment (ablation vs. excision)	79.88	1, 56	<0.001*	0.59
	Dysplasia × treatment	0.80	1, 56	0.375	0.01
	Time	212.77	2, 112	<0.001*	0.79
	Dysplasia × time	0.45	2, 112	0.714	0.01
	Treatment × time	32.21	2, 112	<0.001*	0.37
	Dysplasia × treatment × time	1.01	2, 112	0.390	0.02
Swelling	Dysplasia grade (no vs. mild)	1.60	1, 56	0.211	0.03
	Treatment (ablation vs. excision)	23.55	1, 56	<0.001*	0.30
	Dysplasia × treatment	1.60	1, 56	0.211	0.03
	Time	183.13	2, 112	<0.001*	0.77
	Dysplasia × time	0.78	2, 112	0.509	0.01
	Treatment × time	10.61	2, 112	<0.001*	0.16
	Dysplasia × treatment × time	0.05	2, 112	0.987	0.01

Between-group comparisons at each time point confirmed that ablation was consistently superior to excision during the early recovery period (Table 5). At week 1 and week 3, all four outcomes demonstrated statistically significant differences favoring ablation, with large effect sizes (Cohen's d ranging from 0.70 to 2.25). The differences were most pronounced for bleeding (week 1) and healing (week 1). By week 5, the differences diminished substantially, with only bleeding retaining statistical significance (p = 0.041), whereas healing and pain became non-significant, and swelling showed no difference (p = 0.180).

**Table 5:** Between-group comparison (ablation vs. excision) at multiple time points Healing, pain, and bleeding scores are presented as mean ± SD and were compared using an independent-samples Student's t-test. Swelling scores are presented as median (IQR) and were compared using the Mann-Whitney U test because of non-normal distribution. Effect size is reported as Cohen's d for parametric comparisons and rank-biserial correlation (r) for Mann-Whitney U comparisons. *p < 0.05 values indicate statistical significance. SD: standard deviation, IQR: interquartile range

Outcome	Group	Ablation (n = 30)	Excision (n = 30)	Test value	p-value	Effect size
Healing score (mean ± SD)	Week 1	2.82 ± 0.45	3.60 ± 0.49	t = 6.43	<0.001*	1.66
	Week 3	1.86 ± 0.53	2.22 ± 0.50	t = 2.71	0.009*	0.70
	Week 5	1.19 ± 0.20	1.30 ± 0.31	t = 1.64	0.106	0.42
Pain score (mean ± SD)	Week 1	4.77 ± 0.84	6.29 ± 1.05	t = 6.22	<0.001*	1.61
	Week 3	2.99 ± 0.93	4.11 ± 1.00	t = 4.50	<0.001*	1.16
	Week 5	1.78 ± 0.96	2.22 ± 0.77	t = 1.96	0.055	0.51
Bleeding score (mean ± SD)	Week 1	2.78 ± 1.09	5.32 ± 1.17	t = 8.70	<0.001*	2.25
	Week 3	1.90 ± 0.83	3.16 ± 0.65	t = 6.53	<0.001*	1.69
	Week 5	1.30 ± 0.65	1.69 ± 0.79	t = 2.09	0.041*	0.54
Swelling score (median (IQR))	Week 1	2.0 (1.5-2.25)	3.0 (2.5-3.0)	U = 210	<0.001*	0.62
	Week 3	1.0 (1.0-1.75)	2.0 (1.75-2.0)	U = 265	<0.001*	0.49
	Week 5	1.0 (0.5-1.0)	1.0 (1.0-1.0)	U = 420	0.180	0.17

When stratified by dysplasia grade, ablation demonstrated significantly better outcomes at week 1 across both no-dysplasia and mild-dysplasia subgroups for all parameters (all p < 0.001), with large effect sizes (Cohen's d ranging from 1.49 to 2.44). At week 3, all outcomes remained significantly better with ablation in the no-dysplasia subgroup (p ≤ 0.014), whereas in the mild dysplasia subgroup, only pain (p = 0.026) and bleeding (p < 0.001) remained significant; healing and swelling were comparable. By week 5, no significant differences persisted in either subgroup for any of the outcomes (Table 6).

**Table 6:** Between-group comparison stratified by dysplasia at multiple time points Healing, pain, and bleeding scores are expressed as mean ± SD and were compared using an independent-samples Student's t-test within each dysplasia subgroup. Swelling scores are expressed as median (IQR) and were analyzed using the Mann-Whitney U test. Effect size is reported as Cohen's d for parametric analyses and rank-biserial correlation (r) for nonparametric analyses. *p < 0.05 values indicate statistical significance. SD: standard deviation, IQR: interquartile range

Parameters	Dysplasia	Follow-up	Ablation (n = 30)	Excision (n = 30)	Test value	p-value	Effect size
Healing score (mean ± SD)	No dysplasia	Week 1	2.71 ± 0.54	3.48 ± 0.41	-4.42	<0.001*	1.61
		Week 3	1.63 ± 0.41	2.10 ± 0.50	-2.78	0.010*	1.03
		Week 5	1.15 ± 0.19	1.35 ± 0.38	-1.89	0.073	0.67
	Mild dysplasia	Week 1	2.93 ± 0.35	3.72 ± 0.57	-4.56	<0.001*	1.67
		Week 3	2.09 ± 0.64	2.33 ± 0.49	-1.18	0.250	0.42
		Week 5	1.23 ± 0.21	1.25 ± 0.24	-0.25	0.807	0.09
Pain score (mean ± SD)	No dysplasia	Week 1	4.76 ± 1.06	6.35 ± 1.07	-4.09	<0.001*	1.49
		Week 3	2.81 ± 0.96	4.27 ± 1.07	-3.92	<0.001*	1.44
		Week 5	1.54 ± 0.85	2.10 ± 0.91	-1.75	0.091	0.64
	Mild dysplasia	Week 1	4.78 ± 0.61	6.23 ± 1.03	-4.68	<0.001*	1.71
		Week 3	3.17 ± 0.89	3.95 ± 0.93	-2.34	0.026*	0.86
		Week 5	2.02 ± 1.07	2.34 ± 0.62	-1.00	0.328	0.37
Bleeding score (mean ± SD)	No dysplasia	Week 1	2.80 ± 1.22	5.44 ± 1.12	-6.19	<0.001*	2.25
		Week 3	2.01 ± 1.15	2.88 ± 0.49	-2.71	0.014*	0.98
		Week 5	1.24 ± 0.66	1.62 ± 0.94	-1.26	0.218	0.47
	Mild dysplasia	Week 1	2.75 ± 0.96	5.19 ± 1.21	-6.10	<0.001*	2.23
		Week 3	1.79 ± 0.51	3.43 ± 0.80	-6.68	<0.001*	2.44
		Week 5	1.35 ± 0.64	1.75 ± 0.64	-1.70	0.101	0.63
Swelling score (median (IQR))	No dysplasia	Week 1	2.0 (1.0-2.0)	3.0 (2.0-3.0)	30.00	<0.001*	0.73
		Week 3	1.0 (1.0-1.5)	2.0 (2.0-2.0)	44.00	0.001*	0.61
		Week 5	1.0 (0.0-1.0)	1.0 (1.0-1.0)	84.50	0.140	0.25
	Mild dysplasia	Week 1	2.0 (2.0-2.5)	3.0 (3.0-3.0)	48.00	0.003*	0.57
		Week 3	1.0 (1.0-2.0)	2.0 (1.5-2.0)	75.00	0.083	0.33
		Week 5	1.0 (1.0-1.0)	1.0 (1.0-1.0)	105.50	0.715	0.06

Table 7 showed that within each treatment group, repeated-measures analysis demonstrated significant improvement across all outcomes over the follow-up period (all p < 0.001) with large effect sizes (η²p: 0.51-0.77). Pairwise comparisons revealed that all three time-point comparisons were significant for healing, pain, and bleeding in both groups (all p < 0.001), indicating a steady, progressive improvement. Both groups showed significant improvement in swelling from week 1 to week 3 and from week 1 to week 5. However, in the ablation group, the comparison from week 3 to week 5 was not significant (p = 1.000), suggesting that swelling reached a plateau by week 3. In contrast, the excision group showed a significant reduction in swelling between weeks 3 and 5 (p < 0.001).

**Table 7:** Within-group repeated-measures comparison Repeated measurements of healing, pain, and bleeding scores were analyzed using one-way repeated-measures ANOVA followed by Bonferroni-adjusted pairwise comparisons. Swelling scores were analyzed using the Friedman test followed by a Wilcoxon signed-rank test statistic for post hoc pairwise comparisons. Effect size is reported as partial eta squared (η²p) for repeated-measures ANOVA and Kendall's coefficient of concordance (W) for the Friedman test. *p < 0.05 values indicate statistical significance. SD: standard deviation, IQR: interquartile range, ANOVA: analysis of variance

Outcome	Group	Test value	p-value	Effect size	Week 1 vs. week 3		Week 1 vs. week 5		Week 3 vs. week 5	
					Test value	p-value	Test value	p-value	Test value	p-value
Healing score (mean ± SD)	Ablation (n = 30)	F = 72.8	<0.001*	0.72	13.60	<0.001*	26.16	<0.001*	8.84	<0.001*
	Excision (n = 30)	F = 95.4	<0.001*	0.77	19.71	<0.001*	35.82	<0.001*	14.02	<0.001*
Pain score (mean ± SD)	Ablation (n = 30)	F = 64.9	<0.001*	0.69	14.12	<0.001*	23.20	<0.001*	9.04	<0.001*
	Excision (n = 30)	F = 83.6	<0.001*	0.74	15.01	<0.001*	29.69	<0.001*	14.43	<0.001*
Bleeding score (mean ± SD)	Ablation (n = 30)	F = 29.7	<0.001*	0.51	6.17	<0.001*	10.31	<0.001*	5.51	<0.001*
	Excision (n = 30)	F = 88.2	<0.001*	0.75	13.87	<0.001*	23.77	<0.001*	14.07	<0.001*
Swelling score (median (IQR))	Ablation (n = 30)	χ² = 32.0	<0.001*	0.60	12.71	<0.001*	13.79	<0.001*	0.00	1.000
	Excision (n = 30)	χ² = 127.0	<0.001*	0.71	20.00	<0.001*	29.60	<0.001*	29.80	<0.001*

Within-group analysis stratified by dysplasia grade revealed that all subgroups demonstrated significant improvements over time for all outcomes (all p < 0.001), with effect sizes ranging from 0.38 to 0.75 (Table 8). Pairwise comparisons showed that for healing and pain, all three time-point differences were significant across all subgroups, indicating a sustained improvement throughout the follow-up period. For bleeding, a distinct pattern emerged; in the ablation subgroups, all pairwise comparisons were significant compared to the excision subgroups, suggesting that the ablation groups had more gradual bleeding reduction. For swelling, the ablation subgroups showed no change between weeks 3 and 5, whereas the excision subgroups continued to show a significant reduction (both p < 0.001).

**Table 8:** Intragroup repeated-measures comparison stratified by subgroup Within each treatment and dysplasia subgroup, healing, pain, and bleeding scores were analyzed using one-way repeated-measures ANOVA followed by Bonferroni-adjusted pairwise comparisons. Swelling scores were analyzed using the Friedman test followed by a Wilcoxon signed-rank test statistic for post hoc pairwise comparisons. Effect size is reported as partial eta squared (η²p) for repeated-measures ANOVA and Kendall's coefficient of concordance (W) for Friedman analyses. *p < 0.05 values indicate statistical significance. SD: standard deviation, IQR: interquartile range, ANOVA: analysis of variance

Outcome	Follow-up	Dysplasia	Test value	p-value	Effect size	Week 1 vs. week 3		Week 1 vs. week 5		Week 3 vs. week 5	
						Test value	p-value	Test value	p-value	Test value	p-value
Healing score (mean ± SD)	Ablation (n = 30)	No dysplasia (n = 21)	F = 64.2	<0.001*	0.69	10.81	<0.001*	14.08	<0.001*	6.03	<0.001*
		Mild dysplasia (n = 9)	F = 51.8	<0.001*	0.64	6.96	<0.001*	26.07	<0.001*	6.47	<0.001*
	Excision (n = 30)	No dysplasia (n = 18)	F = 82.4	<0.001*	0.75	14.76	<0.001*	26.86	<0.001*	8.11	<0.001*
		Mild dysplasia (n =12)	F = 73.6	<0.001*	0.72	12.91	<0.001*	21.90	<0.001*	11.46	<0.001*
Pain score (mean ± SD)	Ablation (n = 30)	No dysplasia (n = 21)	F = 49.1	<0.001*	0.63	9.59	<0.001*	16.31	<0.001*	6.94	<0.001*
		Mild dysplasia (n = 9)	F = 39.7	<0.001*	0.58	9.81	<0.001*	13.77	<0.001*	5.73	<0.001*
	Excision (n = 30)	No dysplasia (n = 18)	F = 71.5	<0.001*	0.71	9.73	<0.001*	21.08	<0.001*	10.76	<0.001*
		Mild dysplasia (n =12)	F = 66.9	<0.001*	0.70	11.55	<0.001*	20.29	<0.001*	9.38	<0.001*
Bleeding score (mean ± SD)	Ablation (n = 30)	No dysplasia (n = 21)	F = 18.9	<0.001*	0.41	3.32	0.005*	6.77	<0.001*	3.58	0.003*
		Mild dysplasia (n = 9)	F = 16.8	<0.001*	0.38	5.28	<0.001*	7.90	<0.001*	3.69	0.002*
	Excision (n = 30)	No dysplasia (n = 18)	F = 79.8	<0.001*	0.74	11.64	<0.001*	18.16	<0.001*	7.05	<0.001*
		Mild dysplasia (n =12)	F = 68.5	<0.001*	0.71	7.88	<0.001*	15.00	<0.001*	11.28	<0.001*
Swelling score (median (IQR))	Ablation (n = 30)	No dysplasia (n = 21)	χ² = 18.0	<0.001*	0.56	7.06	<0.001*	6.76	<0.001*	0.00	1
		Mild dysplasia (n = 9)	χ² = 24.0	<0.001*	0.51	7.06	<0.001*	10.47	<0.001*	0.00	1
	Excision (n = 30)	No dysplasia (n = 18)	χ² = 32.0	<0.001*	0.69	5.23	<0.001*	10.46	<0.001*	10.45	<0.001*
		Mild dysplasia (n =12)	χ² = 31.0	<0.001*	0.65	10.47	<0.001*	10.00	<0.001*	10.47	<0.001*

Among patients with no dysplasia, recurrence was observed in one (4.8%) patient in the ablation group and in two (11.1%) patients in the excision group, with no significant difference between treatments (Table 9). Functional disturbance was reported in seven (33.3%) patients treated with ablation and four (22.2%) patients treated with excision, which was also comparable. In patients with mild dysplasia, recurrence was significantly higher in the ablation group, occurring in seven (77.8%) patients, whereas no recurrence was observed in the excision group. Functional disturbance was noted in three (33.3%) patients in the ablation group and five (41.7%) patients in the excision group, without a significant difference. Overall, recurrence was significantly more frequent following ablation than after excision (8 (26.7%) vs. 2 (6.7%)), whereas the incidence of functional disturbance was comparable between the ablation (10 (33.3%)) and excision (9 (30.0%)) groups.

**Table 9:** Recurrence pattern and functional disturbance in study groups (after six months of follow-up) Values are presented as the number of events/total number of patients in the subgroup (percentage). Denominators vary based on the number of patients in each histopathological grade subgroup. Comparisons between the ablation and excision groups were performed using the Pearson chi-square (χ²) test when the expected cell frequencies were adequate and Fisher's exact test when expected cell counts were <5. *p < 0.05 values indicate statistical significance.

Histopathological grade	Outcome	Ablation (n = 30)	Excision (n = 30)	Test statistics	df	p-value
No dysplasia (N = 39)	Recurrence	1/21 (4.8%)	2/18 (11.1%)	Fisher	1	0.845
	Functional disturbance	7/21 (33.3%)	4/18 (22.2%)	χ² = 0.197	1	0.657
Mild dysplasia (N = 21)	Recurrence	7/9 (77.8%)	0/12 (0.0%)	Fisher	1	0.001*
	Functional disturbance	3/9 (33.3%)	5/12 (41.7%)	χ² = 0.007	1	0.935
Overall (N = 60)	Recurrence	8/30 (26.7%)	2/30 (6.7%)	χ² = 4.320	1	0.038*
	Functional disturbance	10/30 (33.3%)	9/30 (30.0%)	χ² = 0.077	1	0.781

## Discussion

The present study compared two diode laser modalities, ablation and excision, in the surgical management of homogeneous oral leukoplakia, stratified according to histopathological grade. At baseline, the two treatment groups were well-matched for age, sex distribution, tobacco use duration, habit type, lesion size, and anatomical site, with no statistically significant differences (Table 1), thereby minimizing the likelihood that demographic or lesion-related confounders substantially influenced the observed treatment effects. Although baseline demographic and clinicopathological characteristics were comparable between the treatment groups, reducing the likelihood that observed differences were explained by measured baseline heterogeneity, the clinician-directed, non-randomized treatment allocation means that residual confounding and selection bias cannot be completely excluded. Therefore, the observed differences in postoperative outcomes and recurrence may be associated with the treatment modality and dysplasia status rather than representing definitive causal effects.

The most consistent finding across nearly all early postoperative parameters, including wound healing, pain, bleeding, and swelling, was the clear superiority of laser ablation over laser excision at weeks 1 and 3, irrespective of the dysplasia status. This pattern is biologically plausible: ablation achieves superficial tissue vaporization with simultaneous thermal sealing of small blood and lymphatic vessels, thereby minimizing intraoperative and postoperative hemorrhage, edema, and pain [16], while excision, even when performed with a laser, involves a deeper, open surgical wound bed with greater tissue disruption, larger raw areas exposed to the oral environment, and an inherently more inflammatory healing course [17]. These observations are consistent with earlier reports highlighting the hemostatic and minimally invasive advantages of diode laser soft tissue surgery [3,4,6].

The three-way mixed ANOVA reinforced this interpretation, showing significant main effects of treatment modality and follow-up time on healing, pain, bleeding, and swelling (all p < 0.001), together with significant treatment × time interactions for all four parameters. This indicated that the rate of symptomatic resolution, not merely the absolute scores, differed meaningfully between ablation and excision, with ablation demonstrating a faster early recovery trajectory that gradually converged with excision by week 5 and was essentially indistinguishable by month 3. This convergence likely reflects the natural resolution of acute inflammatory and reparative processes over time, irrespective of the initial surgical insult, and is consistent with the general wound healing biology of the OM, which typically achieves near-complete re-epithelialization within four to six weeks regardless of the initial defect size when uncomplicated [18,19].

Interestingly, dysplasia grade exerted a significant independent effect only on wound healing (p = 0.004), with non-dysplastic lesions healing faster than mildly dysplastic lesions. Still, it had no significant influence on pain, bleeding, or swelling, nor did it interact significantly with the treatment modality for any parameter. This suggests that the depth and biological behavior of the dysplastic epithelium may subtly slow re-epithelialization independent of the surgical technique used [20] but does not meaningfully alter the patient-perceived symptom burden of pain, bleeding, or swelling, a distinction of practical relevance when counseling patients preoperatively, as clinicians can reasonably reassure patients with dysplastic lesions that their subjective recovery experience is unlikely to differ from that of non-dysplastic cases, even though objective healing may take marginally longer [8,21].

The most clinically consequential finding, however, was the markedly higher recurrence rate following ablation compared with excision, both overall (26.7% vs. 6.7%, p = 0.038) and, most strikingly, within the mild dysplasia subgroup, where recurrence occurred in more than three-quarters of the ablation-treated patients (77.8%) with no recurrence in the excision group (p = 0.001). This finding aligns closely with the biological rationale that ablation, by its superficial vaporizing nature, cannot guarantee complete removal of the dysplastic epithelium extending beyond the visible clinical margin or into deeper rete ridges. In contrast, excision permits a defined three-dimensional margin of clinically normal tissue to be removed en bloc, thereby reducing the likelihood of residual dysplastic cells [2,17]. This interpretation is strongly supported by Vilar-Villanueva et al., who demonstrated in a survival analysis that inadequate extension of ablation margins independently predicts leukoplakia recurrence following CO₂ laser treatment [22], and by Arduino et al.'s recent appraisal of ongoing randomized trial data, which similarly reported a higher recurrence rate with laser ablation (25.9%) than with scalpel excision (15.3%) over an 18-month follow-up [23]. Taken together, these findings should be interpreted cautiously because recurrence was evaluated over a relatively short follow-up period, which may underestimate the true long-term recurrence rate.

Not all recent literature aligns with this pattern; however, these contradictions are instructive. Luo et al.'s network meta-analysis of 11 randomized trials concluded that laser-based therapies overall reduce both recurrence and postoperative discomfort relative to conventional excision [24], which conflicts with our recurrence findings in dysplastic lesions. However, this discrepancy likely reflects the substantial heterogeneity in laser type (CO₂, Er:YAG, diode), margin protocols, and lesion dysplasia grades pooled within that analysis. Similarly, a double-blind RCT found no significant difference in postoperative pain scores between diode laser and scalpel excision at 24 hours, 48 hours, or seven days [20], which contrasts with our finding of significantly lower pain scores following ablation. This divergence may be explained by differences in study design: the RCT compared laser with a cold-steel scalpel rather than laser-to-laser excision, as in the present study, and used a different pain assessment timeline, illustrating that the comparator technique (scalpel vs. laser excision) meaningfully influences perceived postoperative morbidity [20]. Collectively, these contrasting results underscore the persistent heterogeneity and lack of standardized protocols in the oral leukoplakia literature, a limitation emphasized in prior systematic reviews [1,9].

Functional disturbance and recurrence in the non-dysplastic subgroup did not differ significantly between treatment arms, suggesting that for lesions without dysplasia, the choice between ablation and excision may be guided primarily by patient comfort preferences and lesion accessibility rather than oncological concerns, since both modalities appear to offer comparably low recurrence risk in this lower-risk population.

Clinical implications

The findings of the present prospective observational study suggest that a dysplasia-stratified approach may be considered when selecting the appropriate surgical management of homogeneous oral leukoplakia. Diode laser ablation may offer advantages for carefully selected non-dysplastic lesions because of its favorable postoperative recovery profile. In contrast, diode laser excision may be associated with a lower recurrence rate in lesions exhibiting mild epithelial dysplasia. However, these observations should be interpreted in the context of the study's observational design, clinician-directed treatment allocation, and relatively short six-month follow-up period. Therefore, the present findings should be considered hypothesis-generating and require confirmation in adequately powered randomized controlled trials with longer follow-up before definitive treatment recommendations can be made.

Limitations

Several limitations should be considered when interpreting the findings of this study. First, the non-randomized observational design may have introduced residual selection bias and unmeasured confounding despite comparable baseline characteristics between the treatment groups. Second, the relatively small sample size and six-month follow-up period may limit the generalizability of the findings and preclude assessment of long-term recurrence and malignant transformation. Third, postoperative outcome assessment was not blinded, which may have introduced observer bias, particularly for subjective clinical outcomes such as wound healing and swelling. Finally, although all participants received standardized postoperative instructions and adjunctive care, individual adherence to postoperative recommendations, including tobacco cessation, oral hygiene maintenance, and follow-up compliance, was not objectively monitored. Variations in these behavioral factors may have influenced wound healing and recurrence outcomes. Therefore, larger multicenter randomized controlled trials with longer follow-up and blinded outcome assessment are warranted to validate the present findings.

## Conclusions

This study demonstrates that while diode laser ablation provides superior early postoperative outcomes with reduced pain, bleeding, swelling, and faster healing than laser excision, it is associated with substantially higher recurrence rates, particularly in lesions exhibiting mild epithelial dysplasia. These findings advocate for a dysplasia-stratified, individualized surgical approach. Laser ablation may be preferred for non-dysplastic homogeneous lesions where early recovery is prioritized. In contrast, laser excision may be the modality of choice for dysplastic lesions to ensure adequate margin control and minimize recurrence. Long-term surveillance remains essential irrespective of the treatment modality chosen.
